# Neuroprotective Effect of Daidzein Extracted From *Pueraria* lobate Radix in a Stroke Model *Via* the Akt/mTOR/BDNF Channel

**DOI:** 10.3389/fphar.2021.772485

**Published:** 2022-01-14

**Authors:** Meizhu Zheng, Mi Zhou, Minghui Chen, Yao Lu, Dongfang Shi, Jing Wang, Chunming Liu

**Affiliations:** ^1^ The Central Laboratory, Changchun Normal University, Changchun, China; ^2^ College of Life Science, Changchun Normal University, Changchun, China

**Keywords:** daidzein, BDNF, neuroprotection, AKT/mTOR, ischemic stroke

## Abstract

Daidzein is a plant isoflavonoid primarily isolated from *Pueraria lobate* Radix as the dry root of *P. lobata* (Wild.) Ohwi, have long been used as nutraceutical and medicinal herb in China. Despite the report that daidzein can prevent neuronal damage and improve outcome in experimental stroke, the mechanisms of this neuroprotective action have been not fully elucidated. The aim of this study was to determine whether the daidzein elicits beneficial actions in a stroke model, namely, cerebral ischemia/reperfusion (I/R) injury, and to reveal the underlying neuroprotective mechanisms associated with the regulation of Akt/mTOR/BDNF signal pathway. The results showed that I/R, daidzein treatment significantly improved neurological deficits, infarct volume, and brain edema at 20 and 30 mg/kg, respectively. Meanwhile, it was found out that the pretreatment with daidzein at 20 and 30 mg/kg evidently improved striatal dopamine and its metabolite levels. In addition, daidzein treatment reduced the cleaved Caspase-3 level but enhanced the phosphorylation of Akt, BAD and mTOR. Moreover, daidzein at 30 mg/kg treatment enhanced the expression of BDNF and CREB significantly. This protective effect of daidzein was ameliorated by inhibiting the PI3K/Akt/mTOR signaling pathway using LY294002. To sum up, our results demonstrated that daidzein could protect animals against ischemic damage through the regulation of the Akt/mTOR/BDNF channel, and the present study may facilitate the therapeutic research of stroke.

## 1 Introduction

As a common disease, ischemic stroke remains a major cause of mortality and neurological disability worldwide. The World Health Organization (WHO) has reported that around 15 million people suffer from stroke per year globally, making it a serious health issue that is also documented with high relapse rates (Campbell et al., 2019). Currently, the only effective solution to treatment is thrombolysis. However, there are various restrictions which make it fit for only about 5% of all stroke patients. Due to the narrow window for administering rtPA (recombinant tissue plasminogen activator) treatment, only a small percentage of patients receive rtPA treatment during this therapeutic window (4.5 h) after the onset of stroke (Jiang et al., 2021). This has prompted the search for a chemical that could protect neurons from stroke-induced damage by interfering with the biochemical cascade that leads to cell death in the penumbra. Despite more and more studies focusing on the stroke system, there remain few effective therapeutic drugs suited to clinical applications (Zhang et al., 2020a). At present, there are four types of acetylcholinesterase inhibitors used to treat stroke (donepezil, galantamine, rivastigmine, and tacrine) and the agonist (memantine) of one N-methyl- D -aspartate (NMDA) receptor (Cheng et al., 2019). However, drugs can have adverse reactions due to indiscriminate effects on various central and peripheral organs and tissues (Chu, et al., 2019). For this reason, there is a necessity to explore the complementary and alternative therapies with greater effectiveness and less side effect.

**GRAPHICAL ABSTRACT:**
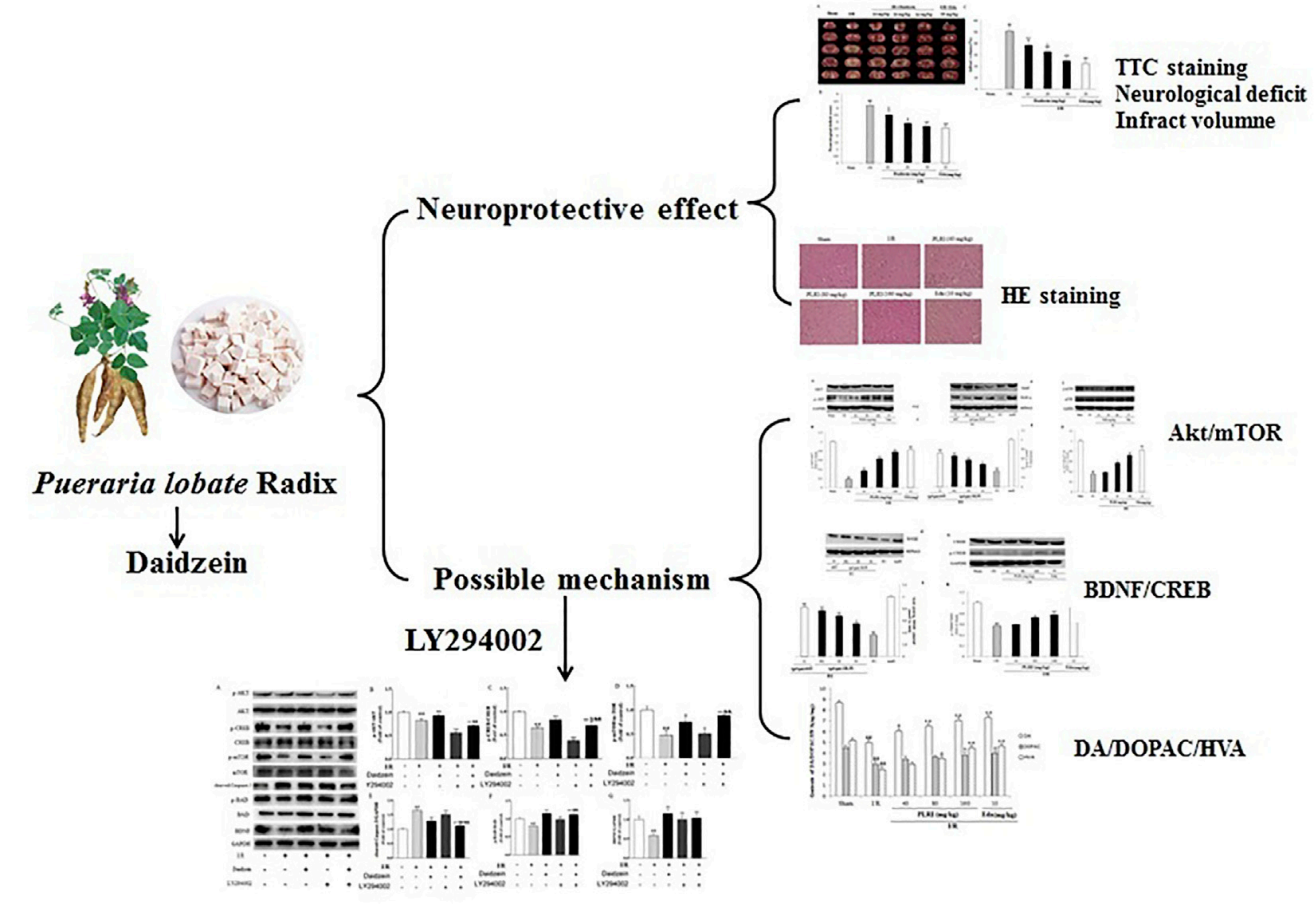


Several mechanisms are found to be involved in cerebral ischemic injury including inflammatory responses, oxidative stress, neuronal apoptosis, excitotoxicity and mitochondrial dysfunction (Wang et al., 2020). So far, it has been suggested that excessive inflammation and immune response are the pathophysiological basis of ischemic brain injury after cerebral infarction (Lee et al., 2021). The phosphoinositide 3-kinase/protein kinase B (PI3K/Akt) signaling regulates various processes including and inflammatory responses, cell growth, survival, metabolism in response to growth factors (Zheng et al., 2017). Previous studies have demonstrated the neuroprotective role of the PI3K/Akt pathway in ischemic stroke models (Xu et al., 2008). P-Akt can activate various downstream proteins, such as Bcl-2 associated death protein (Bad), caspase and so on. Apart from that, brain derived neurotrophic factor (BDNF) can help alleviate cerebral ischemia injury by interfering with apoptotic channels (Zhang et al., 2018). One of the main downstream effector of Akt is mammalian target of rapamycin (mTOR) that exists as 2 multi-protein complexes -mTORc1 and mTORc2 (Ma et al., 2020). The mTOR is a conserved serine/threonine kinase that regulates cell growth and proliferation. The serine/threonine kinase Akt is an upstream regulator of mTOR in mammalian cells, and it is well-known that autophagy is promoted by AKT and inhibited by mTOR (Wang et al., 2019). Accumulating evidence demonstrates that the AKT/mTOR signalling pathway can modulate neuroprotective activation following cerebral ischemia-reperfusion (I/R) (Zheng et al., 2017). For this reason, the AKT/mTOR/BDNF signaling channel is considered to be a potential therapeutic target for treating cerebral I/R impairment.

Daidzein, a monoterpene isoflavone isolated from *P.* lobate Radix, the dry roots of *Pueraria* lobate (Willd.) Ohwi (Radix Pueraria Lobate, Gegen), are officially quoted in the Chinese Pharmacopoeia as antipyretic and spasmolytic agent, as well as the medicine suitable for treating coronary heart diseases, cerebrovascular diseases and hyperlipidemia (Chinese Pharmacopoeia Commission, 2010; Zhang et al., 2020b). According to recently conducted researches revealing the neuroprotective characteristics pertaining to isoflavones, the mentioned nature compounds can potentially mitigate nerve impairment and ameliorate stroke results (Burguete et al., 2016). Daidzein is effective in protecting neural cell from the cell death triggered by oxygen-glucose deprivation by activating receptor-γ under the action of peroxisome proliferator (Hurtado et al., 2012) and in improving poststroke sensorimotor outcomes in mice (Kim et al., 2015). *In vitro*, daidzein protect primary neurons from β-amyloid toxicity (Liu et al., 2012) and from thapsigargin-induced apoptosis (Linford and Dorsa, 2002). In addition, both molecules have been reported to be neuroprotective against glutamate excitotoxicity and oxygen–glucose deprivation (OGD) in cultured neurons (Song et al., 2020). However, the mechanisms of this neuroprotective action have not been fully elucidated.

Edaravone is referred to as one effective antioxidant that moppers free radicals responsible for various neurological disorders. According to the relevant animal research and clinics-related trials, edaravone is effective in protecting neuro from brain diseases (e.g., acute cerebral infarction) (Zheng and Chen, 2016), intracerebral hemorrhage (Dang et al., 2021), Parkinson’s (Li et al., 2021a) and Alzheimer’s disease (Feng et al., 2021), amyotrophic lateral sclerosis (Ortiz et al., 2020) as well as brain trauma (Shakkour et al., 2021). Therefore, Edaravone is treated as positive control herein.

Based on the study of how daidzein could reduce ischemic brain impairment, an investigation was conducted into the role of Akt/mTOR/BDNF signaling channels in mediating the effect of daidzein on cerebral ischemi.We then used LY294002, a PI3K inhibitor, to inhibit the PI3K/Akt/mTOR signaling pathway.

## 2 Material and Methods

### 2.1 Statement of Ethics

IACUC (Institutional Animal Care and Use Committee of Changchun Normal University, Changchun, China) provided the approval for the experiment protocol. Based on international standards on the ethical treatment of animals, all the experimental processes were carried out, and the minimal animal number was adopted for suffering minimization.

### 2.2 Chemicals

We obtained glycine, Tris, TritonX-100, DA, homovanillic acid (HVA), 3, 4-dihydroxyphenylacetic acid (DOPAC), MPTP, dithiothreitol (DTT), sodium dodecyl sulfate (SDS), and LY294002 from Sigma-Aldrich (St. Louis, MO, United States). From our institutional pharmacy, this study acquired Madopar (Shanghai Roche Led., Shanghai, China). Abcam (Dako, Cambridgeshire, United Kingdom) offered Rat anti- glyceraldehyde 3-phosphate dehydrogenase (GAPDH), anti-Akt (total) antibody, rabbit anti-BAD, rabbit anti-cleaved-Caspase-3 antibody, rabbit anti-phospho-Akt (Ser473) antibody, p-BAD, mTOR, p-mTOR, BDNF, CREB, and p-CREB antibodies, horseradish peroxidase (HRP)-conjugated anti-rabbit, and HRP-conjugated rat antibodies. All other chemicals exhibited great-purity analysis level and originated in Shanghai Chemical Reagent Co., Ltd. (Shanghai, China).

### 2.3 Plant Material and Preparation of Daidzein Extracted from *P*. lobate Radix

Tong Ren Tang Medicinal Store (Changchun, China) in 2019 provided *P.* lobate Radix, under the identification by Prof. Shu-MinWang (Changchun University of Chinese Medicine). Voucher specimens (PLP-20–0628) have received the deposition at the Central Laboratory, Changchun Normal University.

**FIGURE 1:**
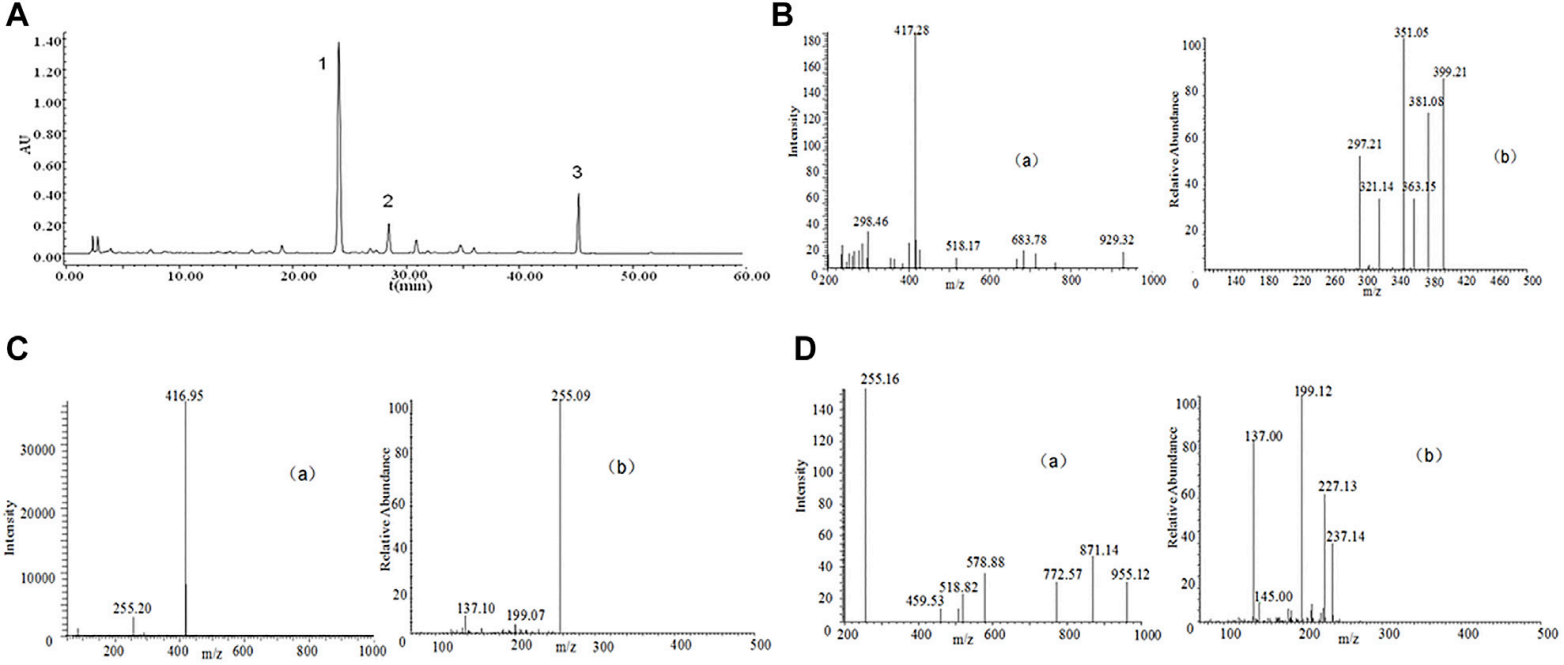
High-performance liquid chromatography (HPLC) chromatograms and total ion chromatogram (TIC) of HPLC-electrospray ionization (ESI)-mass spectrometry (MS) of *P. lobate* Radix extract. **(A)** HPLC chromatograms of *Pueraria lobate* Radix extract **(B)** ESI-MS(a) and secondary mass spectrum of Puerarin (b) **(C)** ESI-MS(a) and secondary mass spectrum of Daidzin (b); **(D)** ESI-MS(a) and secondary mass spectrum of Daidzein (b). HPLC peaks were as follows: 1, Puerarin; 2, Daidzin; 3, Daidzein. Column: C18 column (250 × 4.6 mm id, 5 μm). Column temperature 25°C; mobile phase: consisted of acetonitrile (solvent A) and water containing 0.5% phosphoric acid (solvent B), The *P. lobate* Radix extract was analyzed using the following a gradient program: 0–5 min, 85–85% B; 5–10 min, 85–75% B; 10–20 min, 75–70% B; 20–35 min, 70–55% B; 35–40 min, 55–45% B; and 40–60 min, 45–45% B; flow rate: 0.8 ml/min; and detection wavelength at 250 nm. MS analysis was performed using a DAD instrument by an ESI interface. The mass spectrometer was operated in positive ion modes. The capillary voltage was set at −20 V. The spray voltage was set at 4.5 kV, and the capillary temperature at 250°C.

*P.* lobate Radix (100 g each) received the crushing and 2 h extraction 3 times with reflux inside 70% ethanol under 1,000 ml. The extracts received the separate combination and filtering process via one Whatman #2 filter paper and then the concentration towards dryness with one rotary evaporator under 50°C. Analysis of the compounds in the *P. lobata* Radix extracts was performed on a Waters 2,695 extended by a C18 column (250 × 4.6 mm id, 5 μm), showing its containing 3.83% (w/w) of Puerarin, 1.09% (w/w) of Daidzin and containing 0.36% (w/w) of Daidzein (Figure 1; Table 1). The extraction rate of the *P. lobata* Radix (100 g) was 12.5%.

**TABLE 1:** The MS-MS fragmentation patterns of the isoflavonoids extracted from the *Pueraria* lobate Radix.

Peak	Retention time/min	MS, m/z	MS^2^, m/z	Identified compounds
1	24.2	418.27, [M + H]^+^	351.05, 399.21, 381.08	Puerarin
2	28.7	416.95, [M + H]^+^	255.09	Daidzin
3	45.5	255.16, [M + H]^+^	237.14, 227.13, 199.12	Daidzein

The binary mobile phase comprised water supplemented by 0.5% phosphoric acid (solvent B) and acetonitrile (solvent A). The flow ratio received the maintenance to be constant under 1.0 ml/min. The extracted from *P.* lobate Radix received the analysis with the use of the gradient program below: 0–5 min, 90–80% B; 5–20 min, 80–77% B; 20–35 min, 77–50% B; and 35–40 min, 50–50% B. The authors conducted the monitoring process for the peaks under 250 nm wavelength. Puerarin appeared at 24.2 min, Daidzin at 28.7 min, and Daidzein at 45.5 min (Figure 1).

### 2.4 Animals and Treatment

**FIGURE 2:**
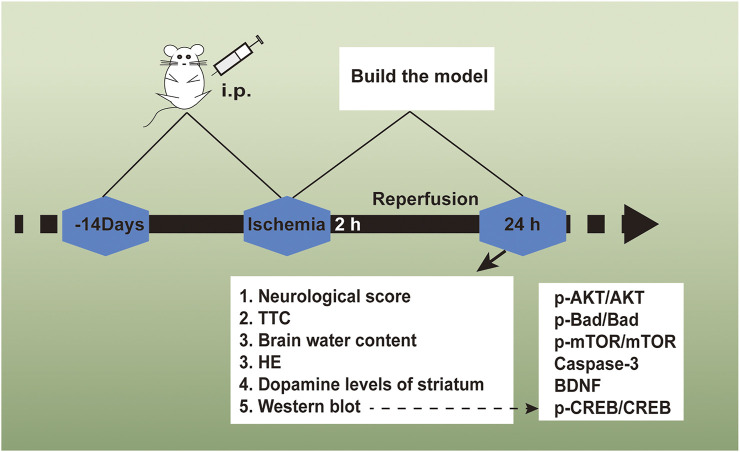
The experimental flow chart of the protective effect exerted by Daidzein in ischemic stroke

The Shanghai Experimental Animal Center, Chinese Academy of Sciences offered ICR rats (male, body weight 25–30 g). 72 adult rats received the random dividing process in 6 cohorts (*n* = 12): the daidzein 10 mg/kg cohort, the daidzein 20 mg/kg cohort, the daidzein 30 mg/kg cohort, the model cohort, the sham cohort, and the Edaravone cohort (10 mg/kg). The drug followed the intragastrical injection one time per day. With presurgery treating process conducted for 2 weeks, stroke received the inducing process inside the rat by I/R, following the previous description, under several modifications (Belayev et al., 1999). In brief, a 5 cm-long nylon filament (0.24–0.28 mm diameter) received the 2 h inserting process in the middle cerebral artery. Sham-operated rats received the identical surgical process as those in the MCAO cohort, with the exception of the middle cerebral artery occlusion. 10 min when ischemia was caused, the rats received the administration with daidzein (10, 20, 30 mg/kg) intraperitoneal injection or the identical normal saline volume (NS). When 2 h was passed, the nylon filament received the careful removal for allowing blood for returning into the ischemic artery. Subsequently, the suturing process was conducted for establishing reperfusion. When the ischemia/reperfusion experiment was completed, the rats were sacrificed, and the brains were immediately removed after the behavioral tests. 5 of them are for TTC, 4 for histopathology, western blot analysis and determination of striatum dopamine levels, 3 for measurement of brain edema. Figure 2 shows the timeline of the experimental flow chart.

#### 2.4.1 Assessment for Neurological Impairments

Prior to the sacrificing process, with Longa’s neurological severity scale, the animal neurological impairments received the evaluation 24 h when the sham or I/R surgery was conducted, following the previous description (Longa et al., 1989). At a 5-point scale, the neurological findings received the scoring: 0, no neurological impairments: 1: failing for overall stretching the fore-limbs and contralateral body; 2: circling for the surgery contralateral side; 3: falling for the surgery contralateral side; 4: depressed consciousness level and lacked spontaneously walking.

#### 2.4.2 TTC Staining and Measuring Process of the Infarct Volume

At the time of neurological impairment evaluation, the rats received the sacrifice under deep anesthesia. For the purpose of TTC staining, 5 rats were randomly selected to comprise the respective cohort. The brains were carefully removed and the sectioning process was carried out in 6 2.0 mm-thick coronal sections. Then, the sections went through the 30 min staining process via 2% TTC in normal saline. Afterwards, the fixing process was conducted in 4% paraformaldehyde solution overnight. The infarct volume was calculated using the infarct size of the six sections multiplied by their thickness. An individual blinded to the treatment carried out all the infarct measuring processes.

#### 2.4.3 Measurement of Brain Edema

The rats were killed 24 h after cerebral ischemia. Then, the brain samples were collected (Zhang et al., 2013). The ischemic area underwent the blotting process with care by using filter paper and then was weighed to obtain wet weights (WW). The ischemic hemispheres were dried for 24 h at 100°C, thus obtaining a constant weight as the dry weight (DW). Water content was calculated using the formula: H_2_O (%) = (WW- DW)/WW × 100%.

#### 2.4.4 HE Staining

A crown zone (2–6 mm after optico chiasm, including hippocampus) of brain tissues was taken as in the embedding box. Then, conventional dehydration, paraffin embedding, slicing (4 μm thickness) and routine HE staining were performed in sequence. Brain tissue lesions were observed under optical microscope (OlympusBX51, Olympus, Japan).

### 2.5 Determination of Striatum Dopamine Levels

The striatum tissue was weighted and homogenized in 200 μl of ice-cold methanol. After 20 min of centrifugation at 14,000 rpm and 4°C, the supernatant was collected and then filtered using a 0.22 μm filter. Then, in accordance with Liquid Chromatography-Mass Spectrometry/Mass Spectrometry (LC-MS/MS), the solution was used to investigate dopamine (DA) and the relevant metabolites (e.g., 3, 4-dihydroxyphenylacetic acid (DOPAC)). LC-MS/MS analyses were conducted as previously described (Zheng et al., 2017).

### 2.6 Western Blot Analysis

When the evaluation was conducted for neurological impairments and TTC staining, the remaining rats’ brains were employed to achieve the Western blotting investigation. The samples received the treating process by using a lysis buffer (0.1% sodium dodecyl sulfate (SDS), 0.5% sodium deoxycholate, 1% Triton X-100, 150 mM NaCl, and 50 mM Tris–HCl (pH8.0)) supplemented by a protease inhibitors cocktail (10 μg/ml leupeptin, 1 mM phenylmethylsulfonylfluo-ride, and2 µg/ml aprotinin). The membranes received the incubation by using major antibodies from the BAD, p-BAD, cleaved-Caspase-3, p-Akt, Akt, mTOR, p-mTOR, BDNF, CREB, p-CREB, and GAPDH proteins (1:1,000) throughout the night at 4°C. When the cleaning process was achieved, blots received the 45 min reaction process by using peroxidase-conjugated secondary antibodies. In addition, based on the enhanced chemiluminescence (ECL) detection system, the protein concentrations received the determination. The staining intensities of the protein bands received the measuring process, quantifying process, and normalizing process against GADPH staining based on the Quantity One software (Bio-Rad Laboratories; Hercules, CA, United States).

### 2.7 To Verify the Effects of Daidzein on the PI3K/Akt Pathway in I/R-Treated Rats

The 60 rats were then randomly divided into 5 groups: sham surgery group, I/R + saline group (rats were administrated with the same volume of physiological saline), I/R + daidzein (rats were treated with daidzein 30 mg/kg), I/R + daidzein + LY294002 group (rats were administrated with both daidzein and PI3K/Akt/mTOR inhibitor LY294002 0.3 mg/kg, Li et al., 2018), and I/R + LY294002 group (rats were treated with LY294002). All animal experiments were approved just as the protocols above.

At the end of the experiment, the rats were sacrificed, and their brains were harvested for Western blotting analysis.

### 2.7 Statistics

All data were expressed as the means ± SD from at least three independent experiments. The data were analyzed by Student’s t test for two group comparisons or one-way analysis of variance (ANOVA), followed by Dunnett’s post hoc test for multiple comparisons, using Graph Pad Prism 6.0 (Graph Pad Software, La Jolla, CA, United States). Differences were considered significant with a *p*-value of less than 0.05.

## 3 Results

### 3.1 Daidzein Down-Regulated Neurological Impairment Scores and Volume of Infarct in Rat After I/R

**FIGURE 3:**
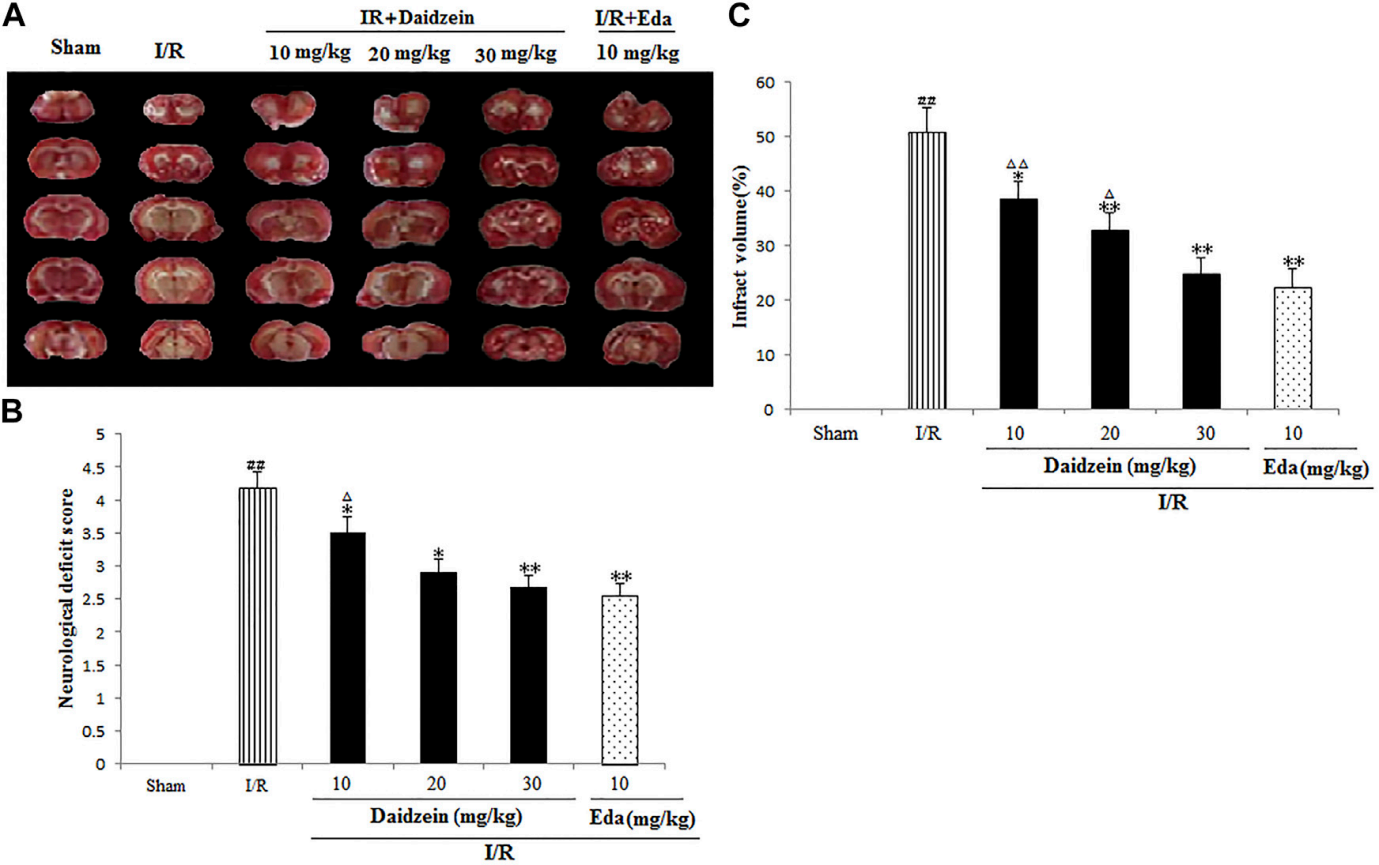
Daidzein attenuated the neurological deficit values and infarct volume of after I/R. **(A)** The influence exerted by daidzein on neurological deficit values **(B)** Representative brain sections stained with TTC; **(C)** The influence exerted by daidzein on the infarct volume. ^##^ *p* < 0.01, in contrast to the Sham group; * *p* < 0.05, ** *p* < 0.05 *vs.* I/R; ^△^ *p* < 0.05, ^△△^ *p* < 0.01, compared with the Eda group.

Neurological impairment was tested against a 5-point scale when ischemic stroke occurred, with higher score indicating greater severity of motor impairment. The results are shown in Figure 3A. The rats in the sham cohort suffered no neurological impairment. Accordingly, a neurological score of zero was maintained throughout the study. Neurological impairment was observed among the I/R cohort (e.g., irregular posture and less spontaneous activity). The aforementioned indicators were improved significantly among the daidzein cohort at 10, 20 and 30 mg/kg (*p* < 0.05, *p* < 0.05, *p* < 0.01) and Edaravone at 10 mg/kg (*p* < 0.01).

Ischemic brain edema was confirmation by the assessment of ischemic brain tissue for its cerebral water content. Brain water content was up-regulated evidently within the ischemia cohort in comparison with the sham cohort (*p* < 0.05). While a reduction to water content was observed in the daidzein cohort (10, 20 and 30 mg/kg) and Edaravone (10 mg/kg) cohort as compared to the ischemia cohort, suggesting that daidzein could reverse the formation of brain edema after ischemic stroke (*p* < 0.05, *p* < 0.01, *p* < 0.01, *p* < 0.01) (Figures 3B,C).

### 3.2 Effect of Daidzein on Pathological Brain Tissue Changes

**FIGURE 4:**
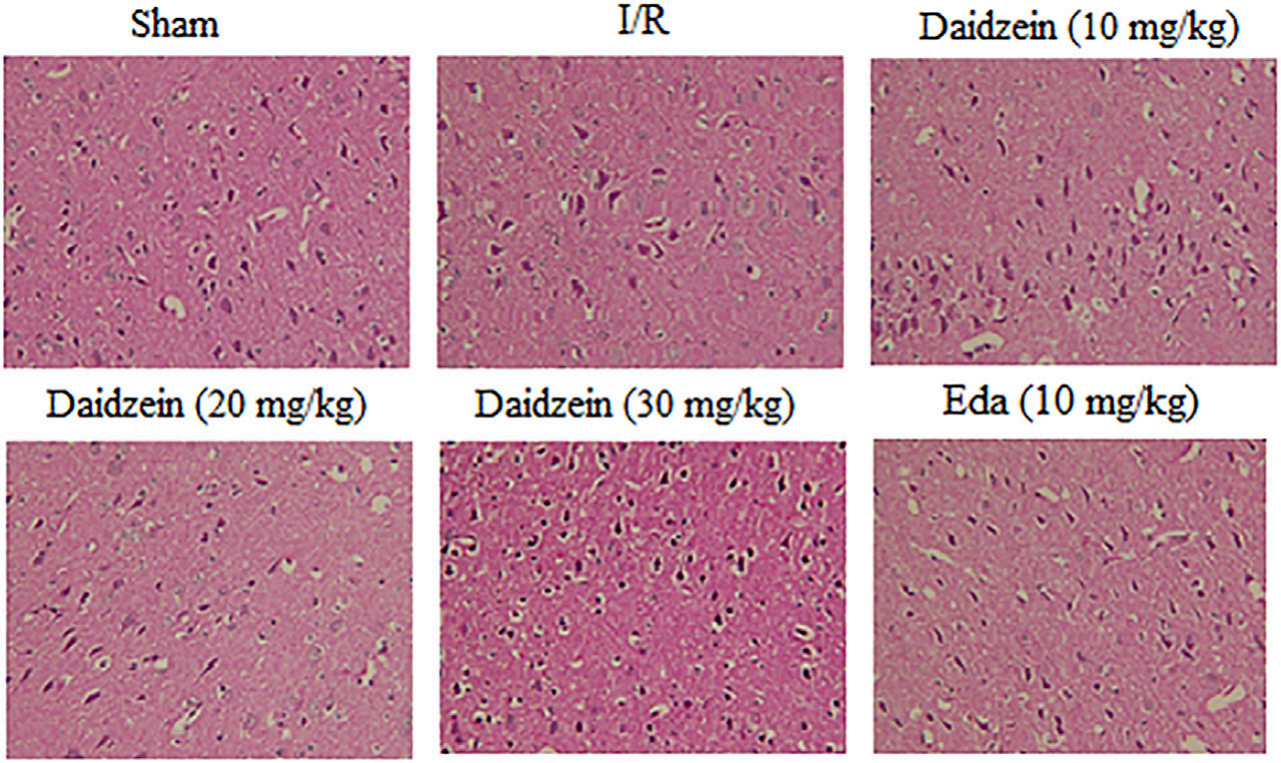
HE staining of daidzein effect upon the ischemic damage after I/R. **(A)** Sham **(B)** I/R **(C)** I/R + daidzein (40 mg/kg) **(D)** I/R + daidzein (80 mg/kg) **(E)** I/R + daidzein (160 mg/kg) **(F)** I/R + Eda (10 mg/kg).

According to HE staining results, there was no abnormality identified in the brain tissue of the sham cohort. To be specific, the neurons were arranged in an orderly way, the morphology was as normal, the nucleolus was clear, and the staining was uniform. The number of cells in the hippocampus was large and the cell level was high. Meanwhile, the cells in the cerebral cortex were abundant and there was no cell contraction observed. There was neither cell edema nor damage detected in the corpus callosum. Among the ischemia cohort, the cells in the hippocampus and cerebral cortex appeared loosened, edema, and contracted. Some brain white matter cells showed significant edema changes, the ischemic area was expanded, the neurons were squeezed. However, the pathological changes of neurons in the ischemic area were significantly reduced after the pretreatment with daidzein at 20 and 30 mg/kg and Edaravone at 10 mg/kg. These results suggest that daidzein contributed to nerve regeneration after traumatic nerve injury (Figure 4).

### 3.3 The Effects Exerted by Daidzein on DA and DA Metabolites within the Rat Striatum

**FIGURE 5:**
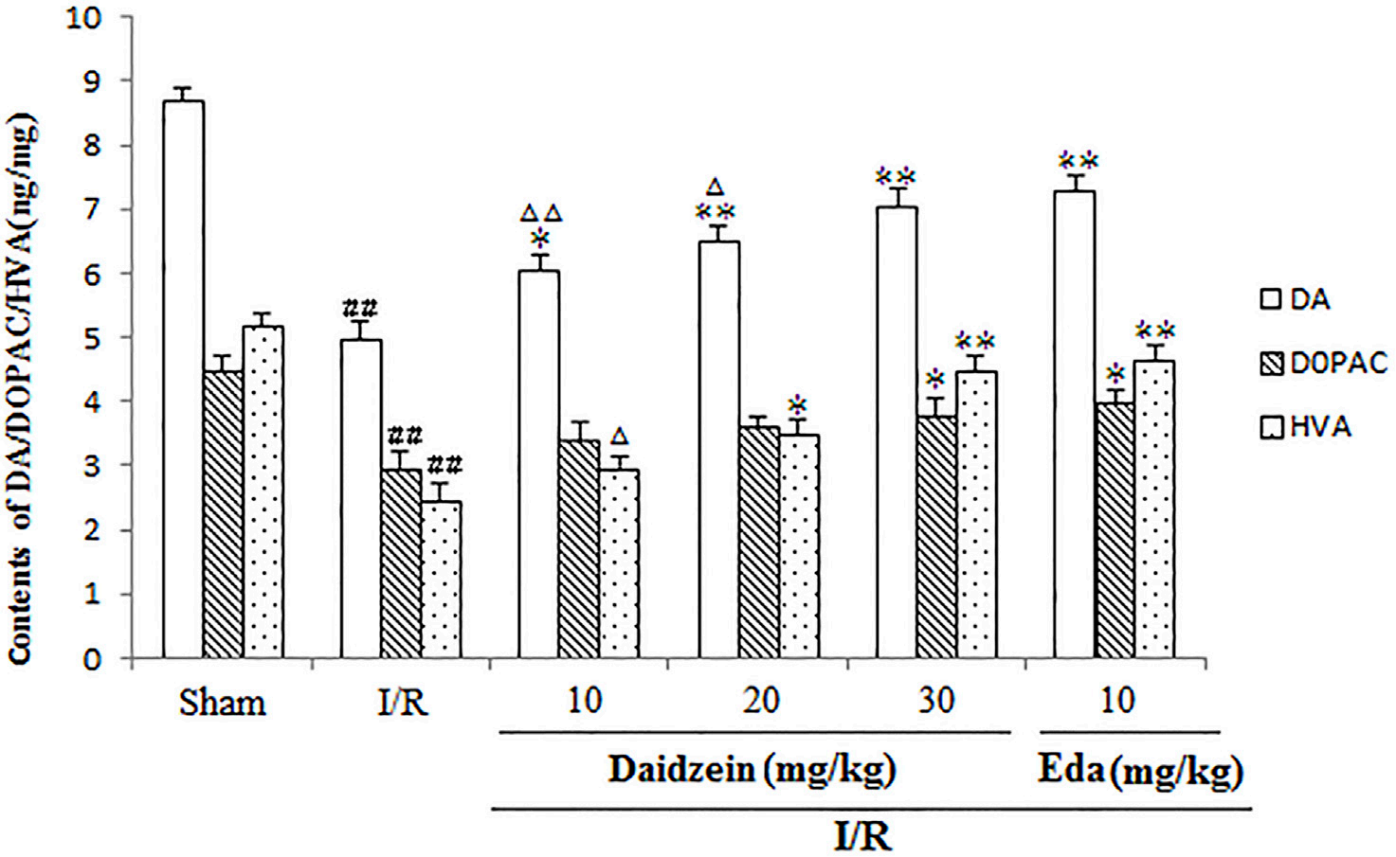
Effects exerted by daidzein on homovanillic acid (HVA), 3, 4-dihydroxyphenylacetic acid (DOPAC), and dopamine (DA) within the striatum prior to I/R. Values have the expression to be means ± standard errors of the means (SEMs; *n* = 6). ^##^ *p* < 0.01, in contrast to the Sham group; *p < 0.05, **p < 0.01, in comparison with the I/R group; ^△^ *p* < 0.05, ^△△^ *p* < 0.01, compared with the Eda group.

Figure 4 shows the results of catecholamine measurement. According to this figure, stroke triggered a sharp decline in dopamine levels and the relevant metabolites as compared to the control (*p* < 0.01, *p* < 0.01, *p* < 0.01). The treatment using daidzein at 30 mg/kg caused the levels of DA, DOPAC and HVA to drop sharply (*p* < 0.01, *p* < 0.05, *p* < 0.01) (Figure 5). This is consistent with the effect of Edaravone at 10 mg/kg (*p* < 0.01).

### 3.4 Effect Exerted by Daidzein on Expression Levels of p-BAD, p-mTOR and p-Akt

**FIGURE 6:**
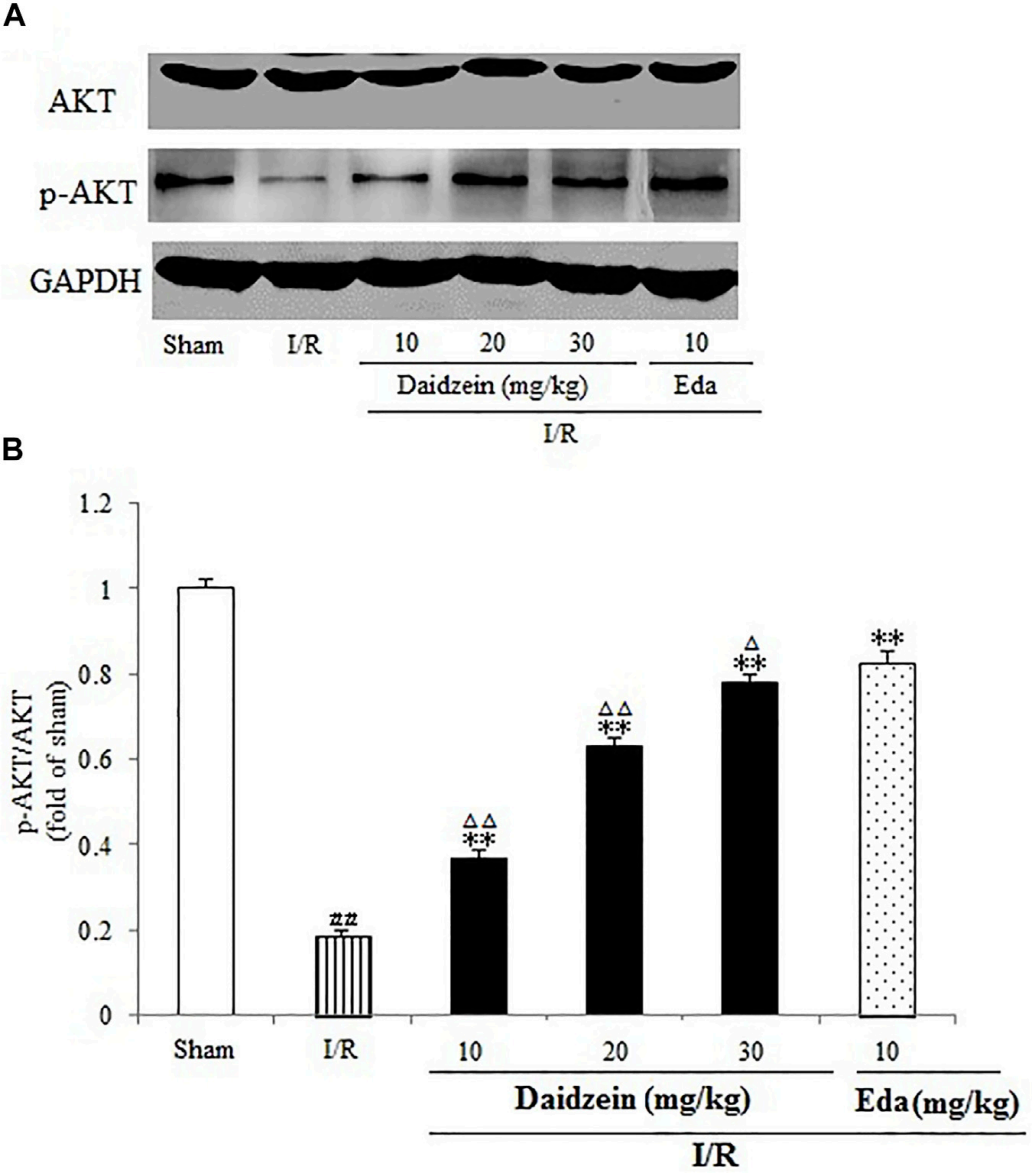
Effects daidzein treatment on the expression of p-Akt and Akt levels in rats’ brain infarct region. **(A)** Representative western blots. **(B)** Ratio of p-Akt/Akt. Values are indicated as means ± standard errors of the means (SEMs; *n* = 3). ^##^ *p* < 0.01 noticeably distinct from Sham; **p < 0.05, noticeably distinct from I/R; ^△^ *p* < 0.05, ^△△^ *p* < 0.01, compared with the Eda group.

**FIGURE 7:**
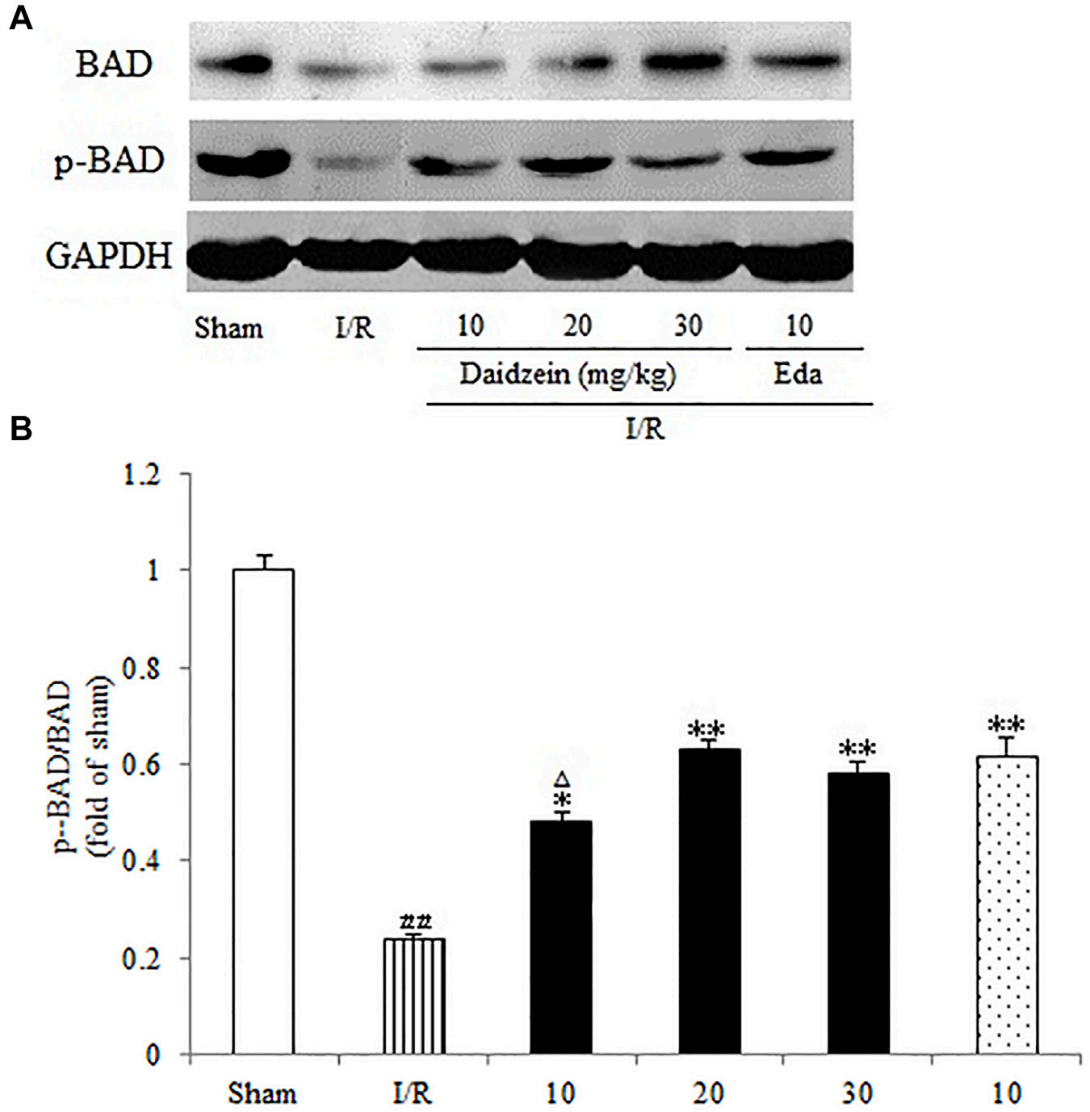
Immunoblotting analysis of protein levels of p-mTOR/mTOR in rats’ brain infarct region. **(A)** Representative western blots. **(B)** Ratio of p-mTOR/mTOR. Values are indicated as means ± standard errors of the means (SEMs; *n* = 3). ^##^ *p* < 0.01 noticeably distinct from Sham; **p < 0.05, noticeably distinct from I/R; ^△^ *p* < 0.05, ^△△^ *p* < 0.01, compared with the Eda group.

**FIGURE 8:**
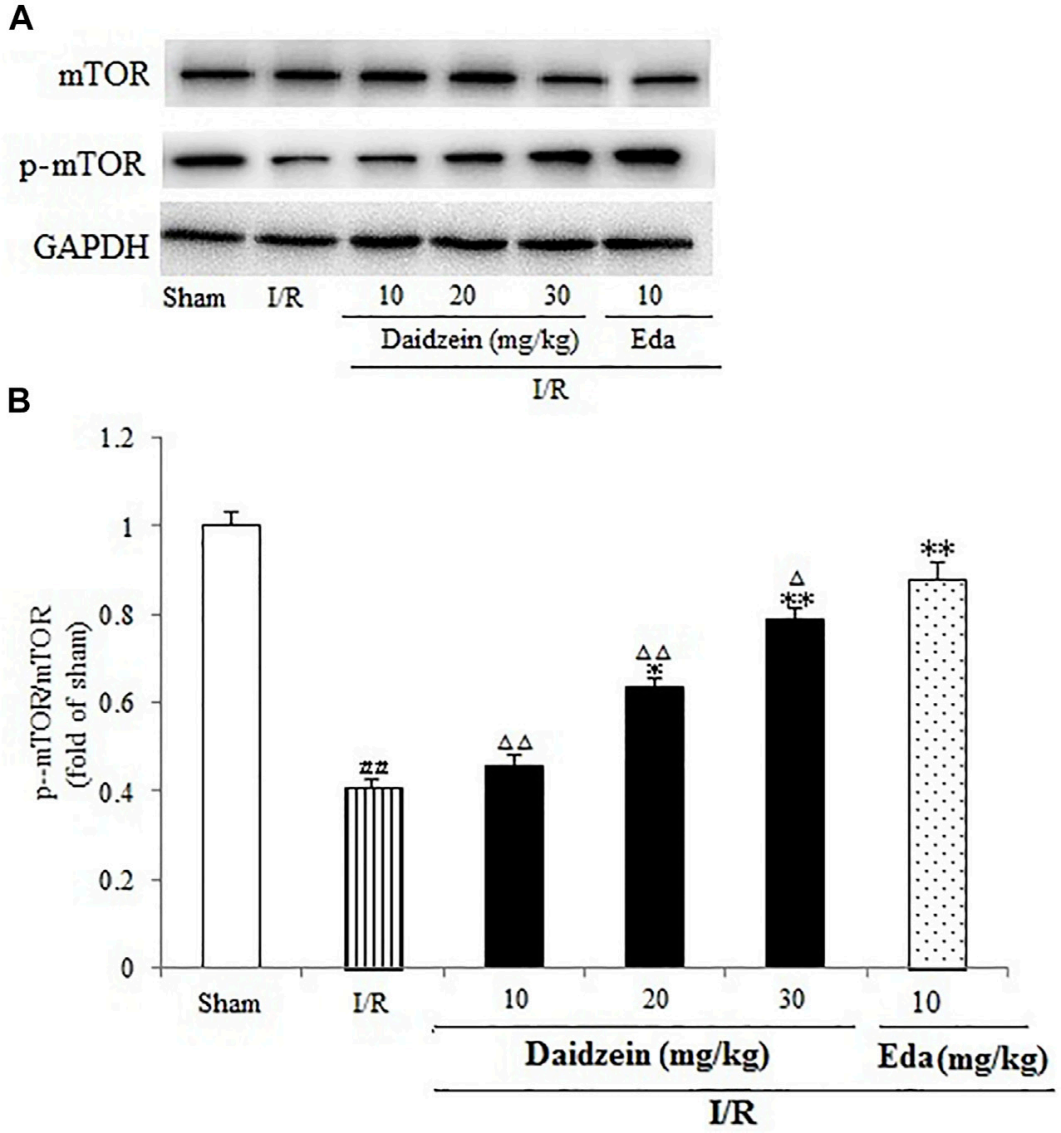
Immunoblotting analysis indicating the Bcl-2/BAD ratio in rats’ brain infarct region. **(A)** Representative protein bands of Bcl-2 and BAD. **(B)** Ratio of Bcl-2/BAD. Values are indicated as means ± standard errors of the means (SEMs; *n* = 3). ^##^ *p* < 0.01 noticeably distinct from Sham; **p < 0.05, noticeably distinct from I/R; ^△^ *p* < 0.05, ^△△^ *p* < 0.01, compared with the Eda group.

Western blotting was performed to estimate the activity of downstream apoptosis-related proteins and PI3K/Akt signaling, as shown in Figures 5–7. As suggested by the results, I/R significantly reduced the expression ration of p-BAD/BAD, p- mTOR/mTOR and p-Akt/Akt as compared to the sham cohort (*p* < 0.01, *p* < 0.01, *p* < 0.01). Relative to the I/R cohorts, the treatment using daidzein (10, 20 and 30 mg/kg) elevated p-BAD/BAD, p-mTOR/mTOR and p-Akt/Akt ratios to a significant extent (*p* < 0.01, *p* < 0.01, *p* < 0.01; *p* < 0.05, *p* < 0.01; *p* < 0.01; *p* < 0.01, *p* < 0.01, *p* < 0.01) (Figures 6, 7, 8).

### 3.5 Daidzein Attenuated Cleaved Caspase-3 Activation in I/R-Treated Mice

**FIGURE 9:**
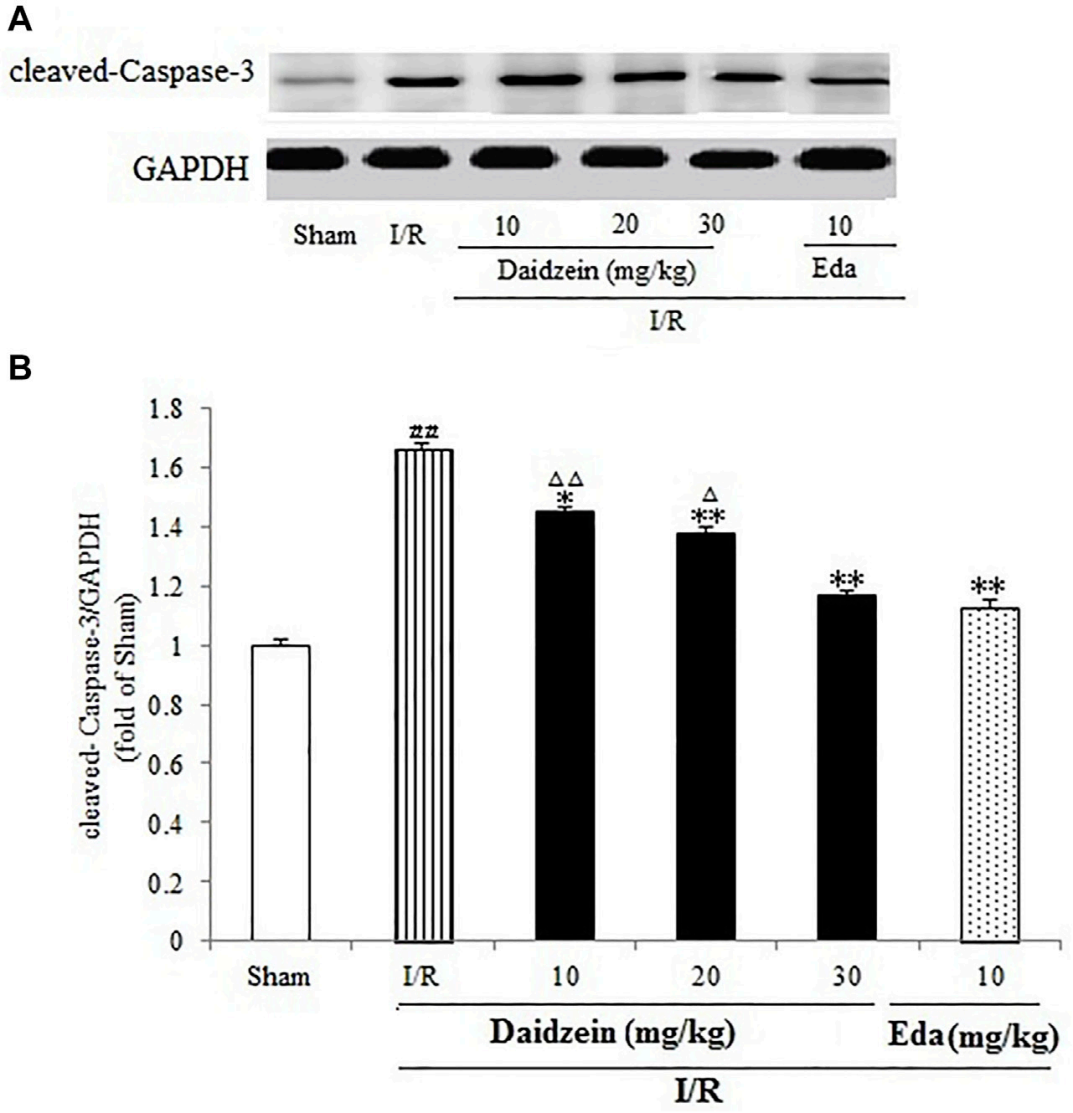
Daidzein treatment decreases the cleaved Caspase-3 level promoted by I/R-treated Rats. **(A)** Immunoblotting analysis of cleavage Caspase-3. **(B)** Ratio of cleaved-Caspase-3/GAPDH. Values are indicated as means ± standard errors of the means (SEMs; *n* = 3). ^##^ *p* < 0.01 noticeably distinct from Sham; **p < 0.05, noticeably distinct from I/R; ^△^ *p* < 0.05, ^△△^ *p* < 0.01, compared with the Eda group.

As shown in Figure 9, I/R treatment enhanced cleaved-Caspase-3 expression significantly in comparison with the sham cohort (*p* < 0.01). Daidzein (10, 20 and 30 mg/kg) preconditioning contributed significantly to reversing the increase of cleaved- Caspase-3 caused by I/R (*p* < 0.05, *p* < 0.01, *p* < 0.01), which was much lower compared with the Edaravone group (*p* < 0.01).

### 3.6 Influence Exerted by Daidzein on the Activation of BDNF/CREB Signaling

**FIGURE 10:**
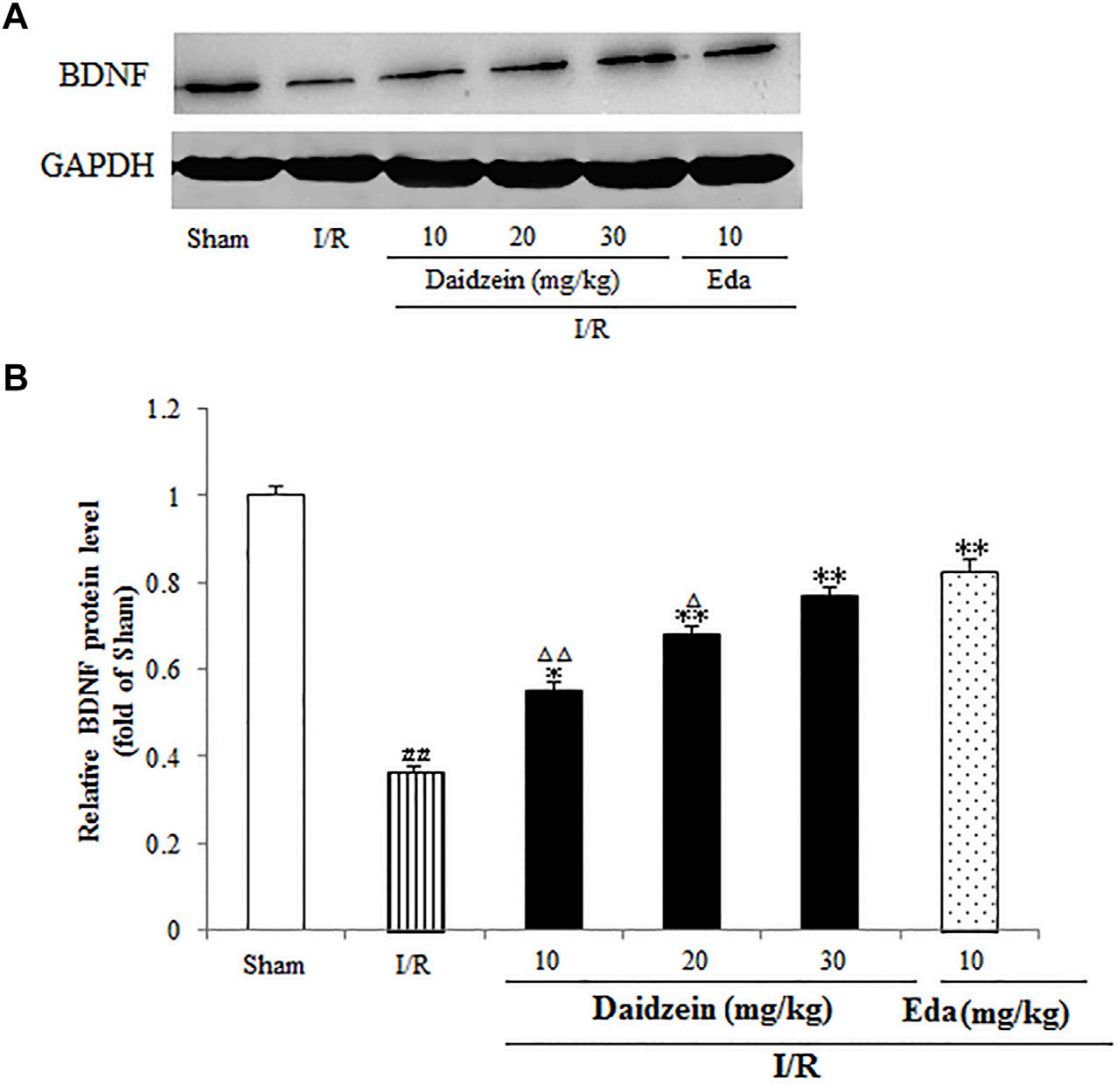
Daidzein conducts the activation for BDNF/Akt/CREB signalling channel for providing neuro-repair in rats. **(A)** Quantifying process of the relative protein extent of BDNF. **(B)** Ratio of BDNF/GAPDH. I/R or daidzein-treatment rats’ brain infarct region. Values are indicated as means ± standard errors of the means (SEMs; *n* = 3). ^#^ *p* < 0.05, noticeably distinct from sham; *p <0.05, noticeably distinct from I/R; ^△^ *p* < 0.05, ^△△^ *p* < 0.01, compared with the Eda group.

A further investigation was conducted into the levels pertaining to CREB and BDNF, which could alleviate the damage caused by ischemia (According to the results, the expression of BDNF was suppressed significantly after I/R surgery (*p* < 0.01). In contrast, it rose sharply after daidzein (10, 20 and 30 mg/kg) and Edaravone at 10 mg/kg treatment was achieved (*p* < 0.05, *p* < 0.01, *p* < 0.01, *p* < 0.01) (Figure 10).

**FIGURE 11:**
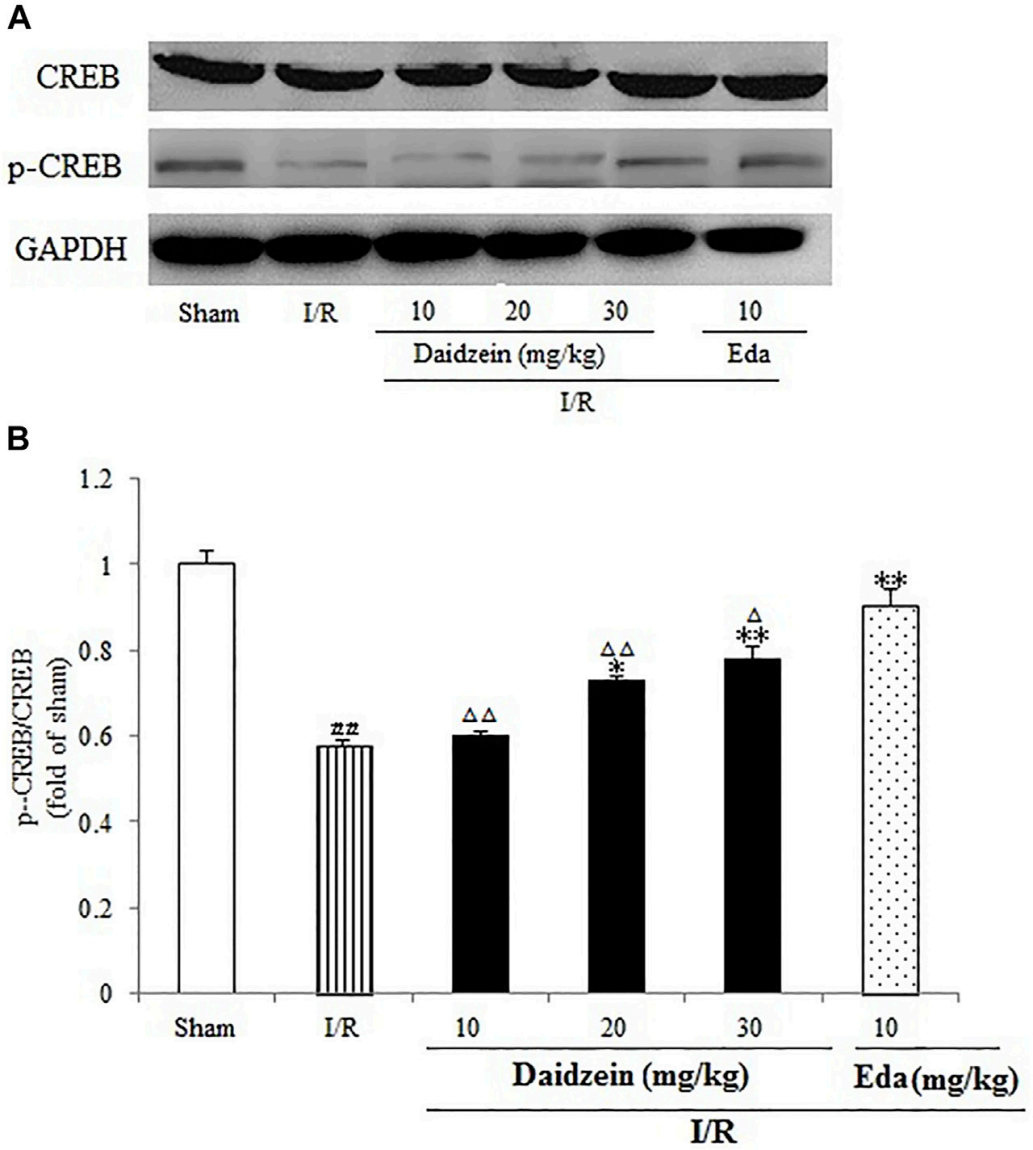
Daidzein conducts the activation for BDNF/Akt/CREB signalling channel for providing neuro-repair in rats. **(A)** Representative protein bands of p-CREB and CREB. **(B)** p-CREB/CREB within the sham, I/R or daidzein-treatment rats’ brain infarct region. Values are indicated as means ± standard errors of the means (SEMs; *n* = 3). ^#^ *p* < 0.05, noticeably distinct from sham; *p <0.05, noticeably distinct from I/R; ^△^ *p* < 0.05, ^△△^ *p* < 0.01, compared with the Eda group.

As shown in Figure 11, I/R surgery reduced p-CREB/CREB rate noticeably, as compared to the sham cohort (*p* < 0.01). However, the decline in p-CREB/CREB rate was significant during the treatment with daidzein at 20 and 30 mg/kg (*p* < 0.05, *p* < 0.01) and Edaravone at 10 mg/kg, in comparison with the I/R cohort.

### 3.7 Confirm the Effects of Daidzein Inhibiting the PI3K/Akt/mTOR Signaling Pathway

**FIGURE 12:**
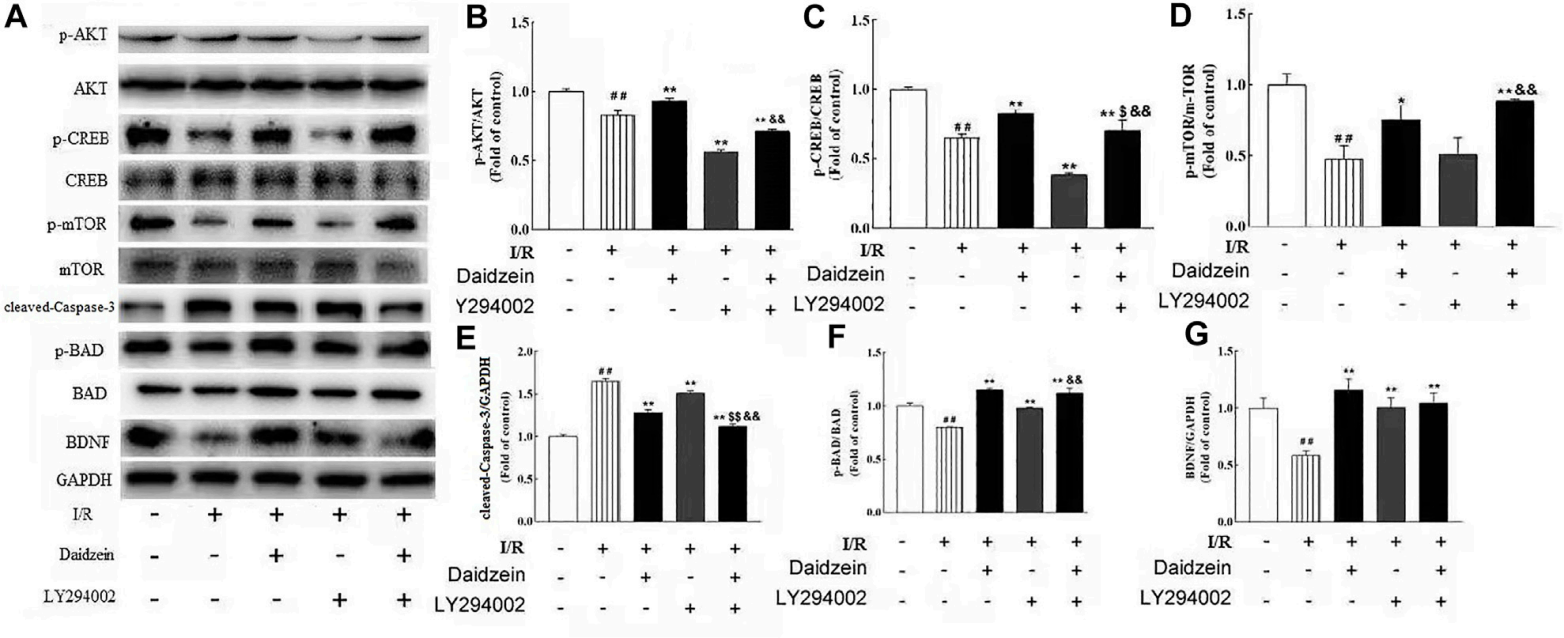
Effect of kinase inhibitors LY294002 on neuroprotection of daidzein against I/R-induced ischemic damage in PIK3/AKT/mTOR pathway. **(A)** Representative western blots. **(B)** Ratio of p-Akt/Akt. **(C)** Ratio of p-CREB/CREB. **(D)** Ratio of p-mTOR/mTOR. **(E)** Ratio of cleaved-Caspase-3/GAPDH. **(F)** Ratio of p-BAD/ BAD. **(G)** Ratio of BDNF/GAPDH. Values are indicated as means ± standard errors of the means (SEMs; *n* = 3). ^##^ *p* < 0.01 noticeably distinct from Sham; **p < 0.05, noticeably distinct from I/R; ^$^ *p* < 0.05, ^$$^ *p* < 0.01, compared to the group treated with I/R+ daidzein; ^&&^ *p* < 0.01compared to the group treated with I/R and LY294002

To further investigate whether the neuro-protective effect of daidzein was regulated by the PI3K/Akt pathway, the PI3K inhibitor LY294002 was used to block the activity of PI3K. Daidzein improved p-Akt, p-mTOR, p-BAD, p-CREB and BDNF levels notably after I/R injury (*p* < 0.01, *p* < 0.01, *p* < 0.01, *p* < 0.01,*p* < 0.01, Figures 12A–G). Notably, Akt, mTOR, BAD and CREB phosphorylation levels and BDNF levels were reduced in the daidzein + LY294002 + I/R group compared with those in the daidzein + I/R group (*p* < 0.01, *p* < 0.01, *p* < 0.01, *p* < 0.01,*p* < 0.01). Moreover, daidzein reduced the cleaved-Caspase-3 level noticeably after I/R injury (*p* < 0.01), while level was improved in the daidzein + LY294002 + I/R group compared with those in the daidzein + I/R group (*p* < 0.01)*.* The above data suggest that daidzein modulated the phosphorylation of downstream targets of the PI3K/Akt pathway, while total Akt and mTOR levels were unaffected (Figure 11).

## 4 Discussion

The aim of this study was at examining the potential mechanism of daidzein against ischemia/reperfusion (I/R). The experiment animal models for stroke were built to simulate the development of cerebral ischemia in humans (Zhou et al., 2021). As revealed from existing research, in the treatment with daidzein at 30 mg/kg per day, corresponding to the habitual intake of isoflavones in Asian adults after conversion (Lee et al., 2021) for 2 weeks, the protection for mice against stroke insult with I/R was conducted consecutively, based on the decrease in the infarct volume and the improvement of neurological impairment. Furthermore, daidzein was demonstrated to activate the PI3K/Akt/mTOR pathway, and LY294002, a specific PI3K inhibitor, significantly reversed the effects of daidzein. The mentioned results suggested daidzein can help alleviate ischemic neuron impairment and prevent I/R-triggered apoptosis, which is attributed at least partially to inhibiting PI3K/mTOR apoptosis channels and activating the BDNF/CREB channel during I/R.

It has been suggested in a number of studies that the dopamine content of extracellular fluid can be significantly increased after cerebral ischemia. Not only could DA produce excitotoxicity, it is also capable to induce the generation of oxygen free radicals, mitochondrial oxidative stress and the accumulation of intracellular calcium ions (Kaushik et al., 2020). As revealed by Li et al., ischemia and reperfusion can cause oxidative metabolites for DA to accumulate in the striatum, which improves the specificity in inducing the generation of oxygen free radicals and inflammatory cytokines (e.g., TNF-a, interleukin (IL)-1b and IL-6), and thus severely damages neurons. Moreover, electro-acupuncture treatment can effectively inhibit the release of DA to protect the brain (Li J et al., 2021). Accordingly, DA is considered to produce neuroprotective effects and promote the restoration of nerve function. As suggested by the results obtained in this study, however, daidzein is capable of reversing the reduction to DA extents and its metabolites, HVA and DOPAC, in the striatum of I/R-treated animals. The mentioned conflicting results may be attributed to the difference between the intra- and interstitial fluids being ignored. The above data suggests the potential of daidzein as a therapeutic option of stroke treatment.

The PI3K/Akt pathway, a major regulator of cell growth and survival, is critical to the mediation of myocardial cell survival in numerous scenarios. Besides, it is responsible for axonal sprouting, which is of high significance to the post-stroke recovery of related functions (Qin et al., 2021). As demonstrated by existing studies, activation of PI3K/Akt can ameliorate I/R injury (Zhang et al., 2020). Furthermore, activation of the PI3K/Akt pathway can phosphorylate mTOR, which has been reported to protect against I/R injury by reducing autophagy and enhancing recovery in the heart (Saiprasad et al., 2014; Meng et al., 2020). Furthermore, the mTOR signal pathway was reported to be highly dependent on the neuroprotective effect in cerebral ischemia (Meng et al., 2020). According to another study, mTOR signal pathway achieved a vital function for protection against ischemic stroke (Li et al., 2021b). Moreover, Ren et al., confirmed that PI3K/Akt/mTOR signaling pathway could protect nerve energy cells by participating in oxidative stress and negatively regulating apoptosis (Ren et al., 2021). Consistent results were achieved here, which demonstrated that daidzein significantly up-regulated the expression levels of p-PI3K, p-Akt and p-mTOR, while LY294002, a specific inhibitor of PI3K, noticeably reversed the aforementioned effects of daidzein. The mentioned findings suggested that daidzein exerted neuroprotection via activating the PI3K/Akt/mTOR pathway.

Existing studies reported that cerebral ischemia down-regulates mTOR as well as Akt, which could prevent BAD translocation in the mitochondrial membrane. As it widely known as the principal mediators of apoptosis, while anti-apoptotic protein BAD could improve the survival of damaged cells by regulating permeabilization of the mitochondrial outer membrane. Though PAKT is capable of inhibiting the transport of BAD to mitochondrial membrane, Akt activation can facilitate the phosphorylation of Bad, thereby inhibiting the apoptotic activity and promoting the survival of cells (Wang A. R et al., 2021). It is already known that the increase of p-Bad contributes to the process of apoptosis inhibition. Apoptosis stimulation can lead to dephosphorylation of BAD, which activates Bax and Caspase-3 (Zhong et al., 2021). Caspase is only activated when caspase is cleaved and initiator caspases, such as caspase-3, are activated. Cleaved Caspase-3 is well known as an executioner protease of apoptosis following brain ischem, and the neuroprotection by resveratrol related to significantly upregulated the expression of p-AKT, p-mTOR, and BCL-2 and downregulated expression of cleaved Caspase-3 and BAX via the PI3K/Akt signaling pathway (Hou, et al., 2018). Consistent with these findings, we demonstrated that I/R increased the levels of cleaved-Caspase-3, while daidzein significantly decreased the expression of cleaved-Caspase-3. LY294002 remarkably (*p* < 0.05) eliminated cleaved-Caspase-3 reduction induced by daidzein.

Furthermore, neurotrophic factors (i.e., CREB and BDNF) can significantly impact the prevention and treatment of ischemic injury (Yang et al., 2018). By interfering with PI3K/Akt apoptotic channels, BDNF could protect against cerebral ischemic injury (Sheikholeslami et al., 2021). Phosphorylated CREB, an activated state of CREB, could up-regulate the expression of BDNF, while BDNF also promotes the activation of CREB through tropomyosin receptor kinase (Trk) B receptors (Zhang et al., 2012; Lan et al., 2014). According to an existing study, the repeated administration of Genistein (a monoterpene isoflavone) was found to improve CREB activity, up-regulate the BDNF expression, and protect the injured nerve of ischemic injury (Xu et al., 2021). Rapid release of BDNF contributed to reverse I/R-induced ischemic injury behaviors in rats (Wang M et al., 2021). Moreover, p-CREB is involved in neuronal apoptosis processes, which is mediated partially by BDNF. p-CREB overexpression inhibited neuronal apoptosis, whereas the inhibition of CREB activity accelerated neuronal apoptosis (Ren et al., 2021).

In this study, an increase in the expression of p-CREB, p-Akt and BDNF was identified after the daidzein treatment, which demonstrated that the BDNF/Akt/CREB signaling channel had a considerable impact on functional recovery after stroke. Despite plenty of researches where Akt and CREB were identified as the downstream targets of BDNF (Massa et al., 2010; Clarkson et al., 2015), CREB is phosphorylated by Akt according to a recent study, causing CREB-mediated expressing state of genes critical for neuronal survival, covering BDNF (Lu et al., 2013). Given the findings made in this study and others, BDNF, Akt, and CREB are suspected to be involved in a regulatory cycle. Plenty of studies indicated that BDNF is released by neurons and is mainly secreted via dendritic release (Zhu et al., 2021; Lee et al., 2020). Given the *in vitro* data used in this study, daidzein enhanced BDNF expression in primary cortical neuron culture (Zhang et al., 2018). In spite of this, how daidzein up-regulates BDNF expression should be investigated in depth, and the direct targets suitable for daidzein therapy should be identified.

To verify the effects of inhibiting the PI3K/Akt/mTOR signaling pathway on stroke model induced by ischemia/reperfusion in rats, LY294002 was intrathecally injected into rats, and the effects were subsequently studied. The results showed that LY294002 significantly alleviated the effect induced by I/R. Moreover, LY294002 significantly down-regulated the expressions of PI3K/Akt/mTOR signaling pathway related factors (Figure 12), which demonstrated that I/R injury was relieved, and that PI3K/Akt/mTOR signaling pathway inhibition might be effective to treat stroke induced by I/R.

## 5 Conclusion

In brief, the results of this study showed that daidzein has therapeutic potential for ischemia/reperfusion-induced brain injury. Although the potential system and feasibility of long-term use remain to be validated, the data still suggests that daidzein promotes neuronal regeneration after ischemic stroke by upregulating Akt/CREB and enhancing BDNF expression. In addition, daidzein may potentially be a new agent to be used in the prevention of focal cerebral ischemia, while also being inexpensive and easily available.

## Data Availability

The original contributions presented in the study are included in the article/Supplementary Materials, further inquiries can be directed to the corresponding authors.

## Abbreviations

AD, Alzheimer’s disease; BDNF, brain-derived neurotrophic factor; BAD, Bcl-2-associated death protein; CREB, cAMP-response element binding protein; DA, Dopamine; DOPAC, 3, 4-dihydroxyphenylacetic acid; DTT, dithiothreitol; ESI, electrospray ionization; GAPDH, glyceraldehyde 3-phosphate dehydrogenase; HE, hematoxylin-eosin staining; HPLC-MS/MS, high-performance liquid chromatography (HPLC)-tandem mass spectrometric (MS/MS); HRP, horseradish peroxidase; HVA, homovanillic acid; I/R, ischemia/reperfusion; NS, normal saline; NMDA, N-methyl-D-aspartate; mTOR, mammalian target of rapamycin; PVDF, polyvinylidene difluoride; PAGE, polyacrylamide gel electrophoresis;PI3K, phosphoinositide 3-kinases; SDS, sodium dodecyl sulfate; TTFA, trifluoracetic acid; TTC, 2,3,5-triphenyltetrazolium chloride.
